# Genome-Wide Analysis of Natural Selection on Human Cis-Elements

**DOI:** 10.1371/journal.pone.0003137

**Published:** 2008-09-10

**Authors:** Praveen Sethupathy, Hoa Giang, Joshua B. Plotkin, Sridhar Hannenhalli

**Affiliations:** 1 Department of Genetics, School of Medicine, School of Engineering and Applied Sciences, University of Pennsylvania, Philadelphia, Pennsylvania, United States of America; 2 Department of Biology, School of Arts and Sciences, School of Engineering and Applied Sciences, University of Pennsylvania, Philadelphia, Pennsylvania, United States of America; 3 Department of Computer and Information Sciences, School of Engineering and Applied Sciences, University of Pennsylvania, Philadelphia, Pennsylvania, United States of America; Indiana University, United States of America

## Abstract

**Background:**

It has been speculated that the polymorphisms in the non-coding portion of the human genome underlie much of the phenotypic variability among humans and between humans and other primates. If so, these genomic regions may be undergoing rapid evolutionary change, due in part to natural selection. However, the non-coding region is a heterogeneous mix of functional and non-functional regions. Furthermore, the functional regions are comprised of a variety of different types of elements, each under potentially different selection regimes.

**Findings and Conclusions:**

Using the HapMap and Perlegen polymorphism data that map to a stringent set of putative binding sites in human proximal promoters, we apply the *Derived Allele Frequency distribution test of neutrality* to provide evidence that many human-specific and primate-specific binding sites are likely evolving under positive selection. We also discuss inherent limitations of publicly available human SNP datasets that complicate the inference of selection pressures. Finally, we show that the genes whose proximal binding sites contain high frequency derived alleles are enriched for *positive regulation of protein metabolism* and *developmental processes*. Thus our genome-scale investigation provides evidence for positive selection on putative transcription factor binding sites in human proximal promoters.

## Introduction

Based on the surprisingly high level of sequence identity between human and chimpanzee proteins, King and Wilson hypothesized that differences in gene regulation underlie the majority of phenotypic variation between these two species[1]. Moreover, it has long been speculated that mutations in gene regulatory elements (GREs) have a significant impact on evolution[2], [3]. Since then, various lines of evidence have confirmed the functional impact of gene regulatory mutations[4].

The majority of known human polymorphisms occur in non-coding regions, many of which are likely to underlie gene expression variation between humans[5]. Moreover, consistent with their potential role in determining phenotypic variability, there is evidence for natural selection acting on specific GREs[6]. However, only recently has it become possible to infer natural selection on entire classes of non-coding elements due to the availability of genome-wide validated single nucleotide polymorphism (SNP) data[7]. Using these datasets, several recent studies have detected selective constraint on conserved non-coding regions[8]–[11]. However, functional non-coding regions are comprised of a heterogeneous mix of elements that may be under different selection regimes. An investigation of natural selection specific to these elements will provide a more detailed view of selection in human non-coding regions. For instance, Chen and Rajewsky have studied natural selection on putative miRNA target sites and found evidence for purifying selection acting on conserved miRNA target sites in the 3′ UTR, and slightly weaker but detectable purifying selection acting on non-conserved miRNA target sites[12]. Here we describe the first genome-scale study of natural selection on transcription factor binding sites (TFBSs) in human proximal promoters, using the *Derived Allele Frequency (DAF) distribution test of neutrality* [*13*]. The DAF test is based on the fact that purifying selection on a derived allele will drive it's frequency towards zero while positive selection will drive it's frequency towards 1. Thus, an overall excess of SNPs with high (respectively low) DAF is indicative of positive (respectively purifying) selection (for an extended description we refer the reader to Supplementary File S1). Using the HapMap SNP dataset we show that the SNPs in human-specific and human-rhesus conserved (i.e., primate-specific) TFBSs have a higher than expected (as defined by comparison to background control SNPs) proportion of derived alleles in the high frequency range. We also provide evidence that this result is robust to TFBS turnover, and is likely not an artifact of SNP ascertainment biases or linkage disequilibrium. Therefore, this result is suggestive of positive selection on recently evolved TFBSs distributed throughout the human genome. Finally, we find that TFBSs that are likely to be undergoing adaptive evolution are enriched in the promoters of genes involved in the regulation of protein metabolism and developmental processes.

## Materials and Methods

### Polymorphism data

We obtained Single Nucleotide Polymorphism (SNP) data from three different sources, the HapMap phase II project (http://www.hapmap.org), Perlegen (http://genome.perlegen.com/browser/download.html), and dbSNP (http://genome.ucsc.edu/cgi-bin/hgTables). The HapMap data provides information on SNPs genotyped in three populations: 90 Yorubans from Ibadan, 90 European-American from central Utah, 44 Japanese from Tokyo, and 45 Han Chinese from Beijing. As is customary, we combined the Japanese and the Chinese populations to form what we referred to as the Asian population. The Perlegen compilation provides information on SNPs genotyped in three populations: African-Americans, European-Americans, and Han Chinese from the Los Angeles area. Approximately 70% of the Perlegen SNPs (referred to as Class A SNPs) were discovered by full re-sequencing of roughly one-third of the human genome in anywhere from 20 to 50 haploid chromosomes. For each population, we retained only those sites identified as biallelic in that population. The total number of SNPs in each dataset is provided in Supplementary File S2. As one of the controls, we downloaded synonymous SNPs from UCSC Table Browser's (http://genome.ucsc.edu/cgi-bin/hgTables) functional annotation of dbSNP build 126. These synonymous SNPs were filtered by selecting only those that were biallelic and genotyped by HapMap in at least one population. Across all populations, there was an average of ∼11,550 HapMap synonymous SNPs. As an additional control, we downloaded the genomic coordinates for introns (excluding the first and last intron) of human protein coding genes from the Ensembl v49 (www.ensembl.org) database for the hg18 genome and identified all of the SNPs that mapped to these regions. Finally, to identify the SNPs that corresponded to the cytosine of a CG dinucleotide, we downloaded the genomic sequences at the three bases centered at each SNP using the Galaxy utility (main.g2.bx.psu.edu).

### Identification of human TFBS

We extracted from Ensembl (http://www.ensembl.org) 1 kb regions upstream of transcription start sites for the 13,003 annotated genes in the Ensembl v49 release (hg18 in UCSC nomenclature) of the reference human genome that have orthologs in rhesus and mouse. Orthology was determined according to the Ensembl v49 release of homology data in the Compara Homology database (http://www.ensembl.org/biomart/martview/). Each polymorphic site in these 1 kb promoter sequences corresponds either to the derived allele or the ancestral allele, and therefore represents only half of the alleles in the human population. Since binding sites are predicted on the basis of sequence content, a site may be predicted in the reference allele and not in the other allele and vice versa. Therefore, to obtain the full set of binding sites that cover both ancestral and derived alleles, we generated another set of 1 kb regions representing the allelic complement of the reference set. We refer to each set of 1 kb regions as *reference* and *allelic_complement*, respectively. For example, consider a reference sequence *ATCGAGT* and suppose there is a known C/G polymorphism at position 3. The allelic complement would be *ATGGAGT*. If there are multiple SNPs within a single TFBS, then it would be preferable to generate exhaustively all allelic complements. However, in practice, this is extremely rare (in all cases, less that 5% of SNPs cluster within a promoter). Therefore, it is reasonable to consider only one allelic complement for the entire 1 kb proximal promoter sequence.

We identified binding sites based on 584 positional weight matrices (PWM) for vertebrate transcription factors in TRANFAC v10.2[14]. However, because many of these PWMs are highly similar, we first clustered the PWMs in 235 classes. The clustering was done using a previously described approach that is based on an information-theoretic measure of pair-wise PWM similarity[15]. Using a previously described tool – PWMSCAN[16], we searched for stringent (p-value≤0.00002) matches of these 235 representative PWMs on both the *reference* and the *allelic_complement* 1 kb promoter regions, and merged the overlapping matches. This p-value threshold corresponds to an average expected frequency of 1 match every 50 kb of human genome. The identified matches are considered putative binding sites and provide the foreground (F) for our analysis.

Predicted foreground (F) TFBSs were partitioned into three subsets: (1) sites that are conserved among human, rhesus, and mouse (HRM), (2) sites that are conserved between human and rhesus (HR) and (3) sites that are predicted in human but neither in rhesus nor in mouse (H). The sets H, HR, and HRM were determined as follows. We downloaded from Ensembl (http://www.ensembl.org) the rhesus and mouse 1.5 kb promoter regions of the orthologous genes (the additional 0.5 kb sequence was used to accommodate for alignment gaps). We then defined ‘conservation’ in two different ways. Consider a predicted TFBS *T* for PWM *M* in the promoter *P* of human gene *G*.

In the first method, we first aligned *P* with the promoter region of *G*'s ortholog in species *S* (rhesus or mouse) using the Needleman-Wunsch algorithm (gap open penalty = 10 and gap extension penalty = 0.5). If PWMSCAN predicts a TFBS for *M* exactly at the aligned region in *S*, then we set *T* as conserved between human and *S*. We refer to this method as *Poscons* because it requires exact positional conservation.

In the second method, if PWMSCAN predicts a TFBS *T* anywhere in the promoter region of *G*'s ortholog in *S*, then we set *T* as conserved between human and *S*. We refer to this method as *Turnover* because it accommodates for the possibility of binding site turnover[17]. In this approach, it is not clear how to deal with the scenario where there are more non-overlapping instances of *T* in the human promoter than in *S*'s promoter (given that *S*'s promoter has at least one instance). Because it is usually unknown which of the instances have “turned over”, we chose to designate all such instances as conserved. The summary statistics of human TFBS predictions according to both the *Poscons* and the *Turnover* methods are given in Tables 1 and 2.

**Table 1:** Summary statistics of predicted TFBSs according to the *Poscons* method.

	# unique binding sites	# nucleotides	average nts/site
**H**	145280	1626723	11.2
**HR**	75303	868618	11.5
**HRM**	9081	96132	10.6

**Table 2:** Summary statistics of predicted TFBSs according to the *Turnover* method.

	# unique binding sites	# nucleotides	average nts/site
**H**	119996	1397972	11.7
**HR**	73622	893119	12.1
**HRM**	36932	410451	11.1

### Information-content approach to determine PWM core

A column in a PWM is a probability distribution of the 4 nucleotide bases, A, C, T and G. Information content (IC) of a PWM column measures its specificity[18]. IC values range from 0 to 2, with 0 indicating no information or specificity and 2 indicating maximum specificity. For instance, a PWM column in which all 4 bases are equally probable has an IC of zero and a column where only one of the 4 bases occurs has an IC of 2[18]. The “core” region of a binding site is defined as a contiguous stretch of nucleotides that is most important for TF-DNA binding. The IC value at a PWM column is an estimate of how important that position is for TF-DNA binding. We determined the core region of each PWM by calculating the cumulative IC for each window of length 5 bp and identifying the window with the highest cumulative IC.

### Poisson random field model

Sawyer and Hartl provided a mathematical framework - the Poisson Random Field (PRF) model, with which to infer the strength of selection on a particular gene[19]. We applied this model to the derived allele frequency distributions of all categories of TFBS and synonymous sites, yielding maximum-likelihood estimates of the mutation rate and selection pressure. For a more detailed presentation of the biological assumptions and mathematical framework of the PRF model, we refer the reader to [19]–[21] and Supplementary File S1.

## Results

### Qualitative inference of selection via DAF distribution analysis

**Figure 1:**
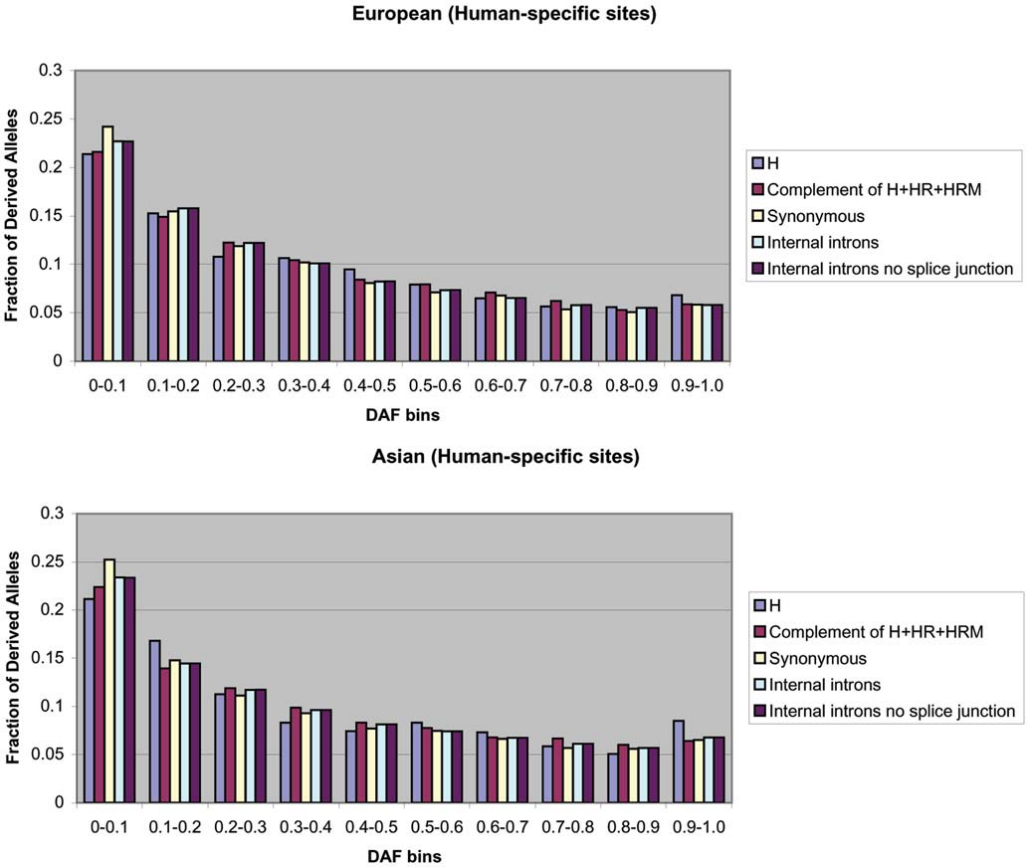
European-American and Asian DAF distributions for human-specific binding sites. H sites were predicted according to the *Poscons* method. H sites have a significantly larger fraction of high frequency derived alleles (defined initially as derived alleles with frequency >90%) than C sites (Fisher's exact test, [European-American] P = 0.088, [Asian] P = 0.002), synonymous sites (Fisher's exact test, [European-American] P = 0.072, [Asian] P = 0.003), and internal introns (Fisher's exact test, [European-American] P = 0.054, [Asian] P = 0.005).

**Figure 2:**
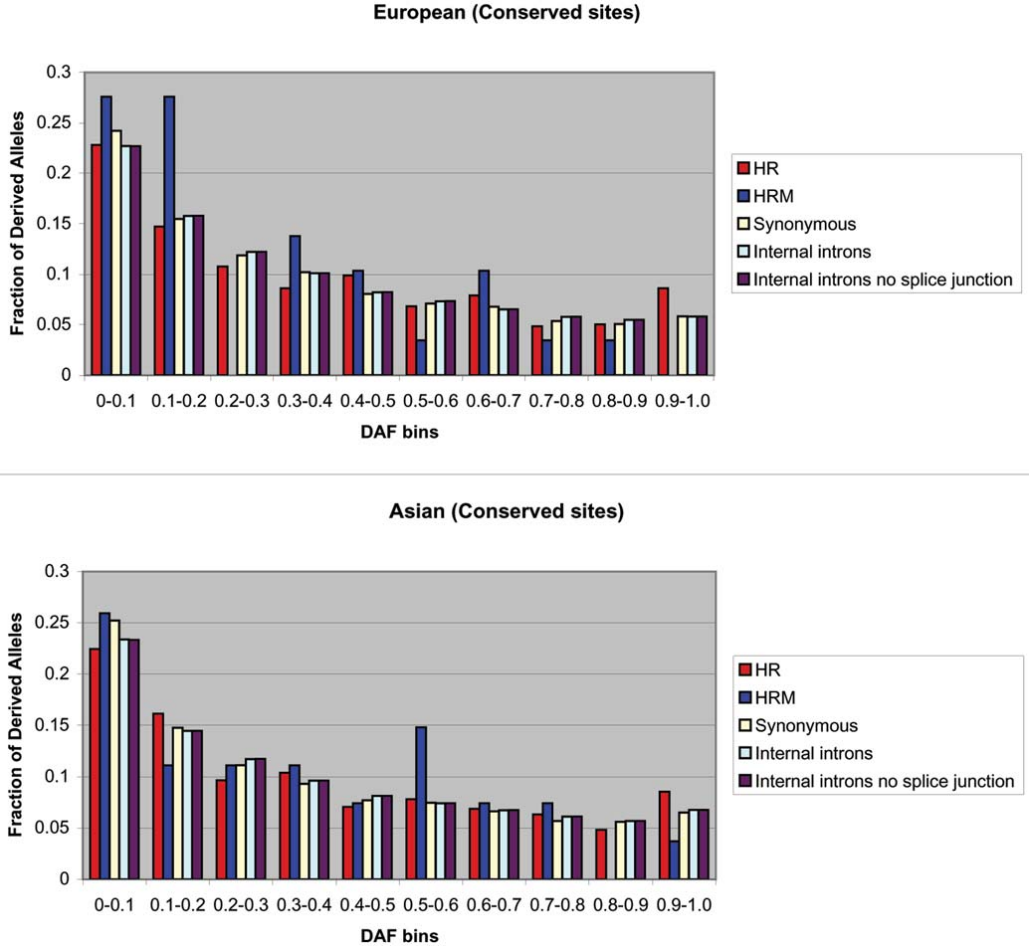
European-American and Asian DAF distributions for human-rhesus conserved and human-rhesus-mouse conserved binding sites. HR and HRM sites were predicted according to the *Poscons* method. HR sites have a significantly larger fraction of high frequency derived alleles than synonymous sites (Fisher's exact test, [European-American] P = 0.006, [Asian] P = 0.043) and internal introns (Fisher's exact test, [European-American] P = 0.005, [Asian] P = 0.065).

Given that SNP density is not informative for the strength and sign of selection[12], we analyzed the derived allele frequency (DAF) distributions, which do not depend on mutation rate. Relative to neutral expectation, a shift in a DAF distribution toward low frequency alleles is indicative of negative selection [11]–[13] and a shift toward high frequency alleles points to positive selection[13], [22]. For each HapMap SNP, using the human-chimp genome-wide alignment files provided by the UCSC Genome Browser, we defined the human derived allele as the allele that differs from the chimpanzee allele at the same locus. If the chimpanzee allele did not match either of the human alleles, then the SNP was discarded from the analysis. For each population, we computed the DAF distribution in three classes of predicted TFBSs according to the *Poscons* method (H, HR, and HRM). Additionally, we mapped HapMap SNPs to, and computed the DAF distribution for, three independent sets of control sites that are intended to approximate neutrally evolving regions: (1) genome-wide set of human synonymous coding sites (S), (2) genome-wide set of internal introns (I) and (3) regions within all of the 1 kb promoters that did not overlap with the predicted TFBSs (C). Selection on synonymous sites is generally weak, and therefore silent sites are often used to approximate selectively neutral DNA[23]. Intronic regions have also been used as a proxy for neutrally evolving DNA[24]. In order to exclude as much functional DNA as possible, we removed first and last introns (which often contain transcription factor binding sites), and the first and last 50 nucleotides of internal introns (which harbor splice junctions and mirtrons). Finally, we also assume that C sites are under minimal selection pressure, since they are presumed to be non-functional regions. Because the HapMap project applied disparate ascertainment conditions to different SNPs, we chose not to compare the DAF distributions of foreground sites with neutral models such as Tajima's D or Fay and Wu's H, following the precedent of[12]. Instead, as in[11], [12], for each population we compared the DAF distribution of the foreground classes of sites directly with the three control classes of sites (all assumed to be evolving neutrally). As shown in Figure 1 and supplementary file S2, we found that in the European-American and Asian populations, the H sites have a significantly larger fraction of high frequency derived alleles (defined initially as derived alleles with frequency >90%) relative to C sites (Fisher's exact test, [European-American] P = 0.088, [Asian] P = 0.002), relative to S sites ( [European-American] P = 0.072, [Asian] P = 0.003), and relative to I sites ([European-American] P = 0.054, [Asian] P = 0.005). And as shown in Figure 2 and supplementary file S2, we also found that in the same two populations, the HR sites have a significantly larger fraction of high frequency derived alleles relative to S sites ([European-American] P = 0.006, [Asian] P = 0.043) and relative to I sites ([European-American] P = 0.005, [Asian] P = 0.065). Further, as shown in supplementary file S2, we found that the fraction of high frequency derived alleles in the combined set of H and HR sites was significantly larger (at 1% significance level) relative to the C sites ([European-American] P = 0.008, [Asian] P = 0.002), the S sites ([European-American] P = 0.005, [Asian] P = 0.002) and the I sites ([European-American] P = 0.002, [Asian] P = 0.004). To ensure the robustness of the result, we performed 1000 independent bootstrap experiments and also repeated the analysis using varying thresholds of “high frequency” (90% and 85%) and still observed similar results (Supplementary File S2).

One potential source of bias is the Cytosine to Thymine hypermutability at methylated CpG dinucleotides which can lead to a mis-inference of the ancestral allele, thus affecting the DAF distribution. In particular, at an XG site where X is a SNP with T as the ancestral allele and C as the derived allele (using chimpanzee as the outgroup), it is possible that in fact C is the ancestral allele and there was a C to T mutation in the chimpanzee lineage. The utilization of chimpanzee as the outgroup species should alleviate this problem to some extent because its close relation to human limits the frequency of mutations at a site along both human and outgroup lineages. Nonetheless, excluding sites of the form XG (where X is defined as above) does not qualitatively change our results (data not shown).

Finally, although we notice that in the European-American and Yoruban populations the HRM sites have a substantially larger fraction of low frequency derived alleles (defined as derived alleles with frequency<20%) than S and I sites, the difference is not statistically significant, possibly because of a lack of power due to a considerably smaller number of SNPs in HRM sites (this is also evident from the fact that the HRM DAF distribution is not smooth). To test this hypothesis, we first applied a less stringent version of *Poscons* TFBS prediction (p-value corresponding to 1 hit every 25 kb), which increased the number of SNPs in HRM sites by a factor of ∼1.8 in each population. We then repeated the DAF analysis on this set of predictions (Supplementary File S2) and found that in the European-American and Yoruban populations, the HRM sites have a statistically significantly larger fraction of low-frequency derived alleles relative to S ([European-American] P = 0.0096, [Yoruban] P = 0.0455) and I sites ([European-American] P = 0.0056, [Yoruban] P = 0.0299). We did not observe this result in the Asian population. If changes in population size and migratory patterns are assumed to be negligible, which in any case should have unbiased effects on the foreground sites relative to the control sites (since they are interleaved throughout the genome), these results may be suggestive of positive selection on both H and HR TFBSs.

**Figure 3:**
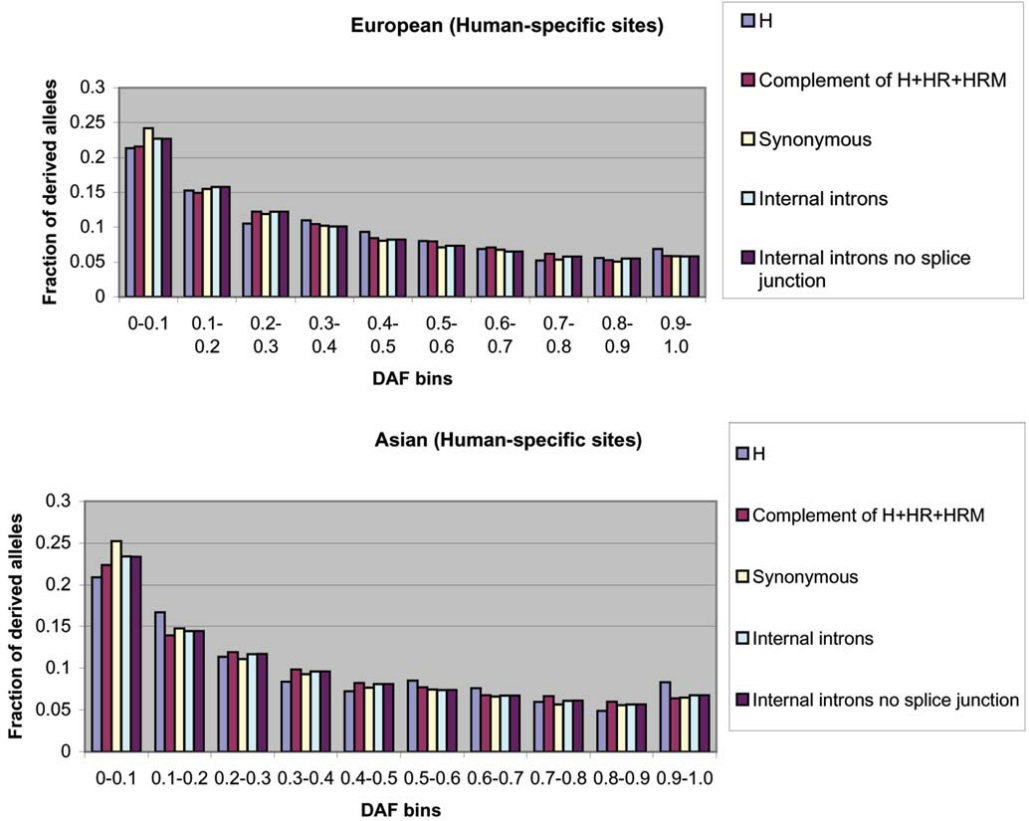
European-American and Asian DAF distributions for human-specific binding sites using the *Turnover* method. H sites have a significantly larger fraction of high frequency derived alleles (>90%) than C sites (Fisher's exact test, [European-American] P = 0.078, [Asian] P = 0.006), synonymous sites (Fisher's exact test, [European-American] P = 0.063, [Asian] P = 0.007) and internal introns (Fisher's exact test, [European-American] P = 0.048, [Asian] P = 0.014).

**Figure 4:**
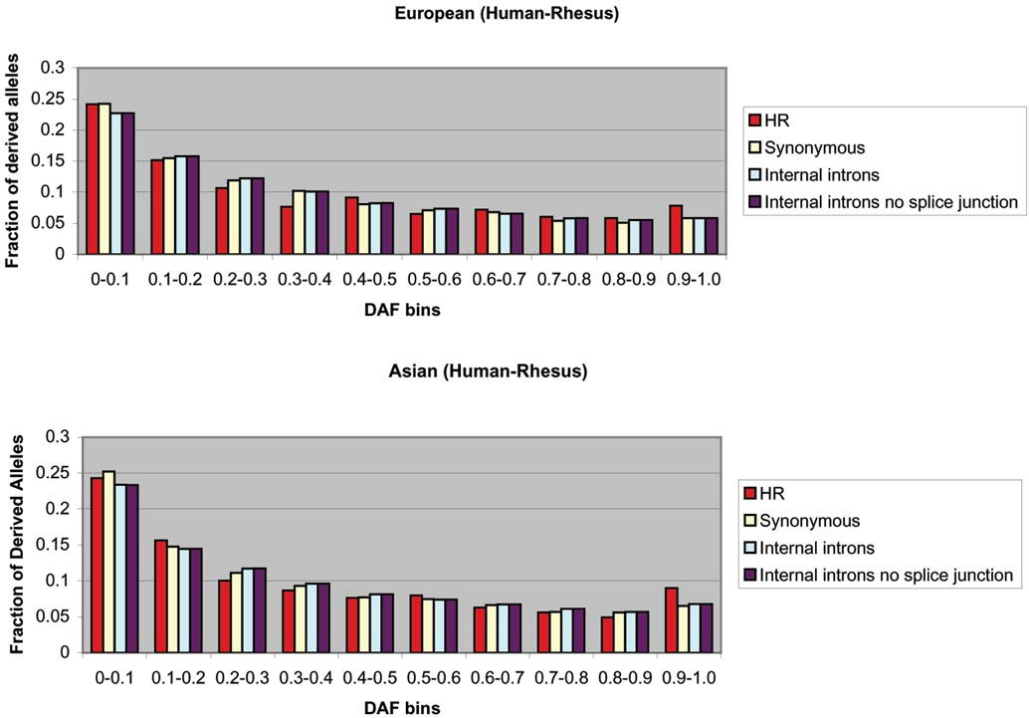
European-American and Asian DAF distributions for human-rhesus conserved binding sites using the *Turnover* method. HR sites have a significantly larger fraction of high frequency derived alleles (>90%) than synonymous sites (Fisher's exact test, [European-American] P = 0.031, [Asian] P = 0.014) and internal introns (Fisher's exact test, [European-American] P = 0.026, [Asian] P = 0.023).

We repeated the above analysis for TFBSs predicted using the *Turnover* method, which allows for the well-known evolutionary phenomenon of binding site turnover[17]. The results were consistent with those from the analysis using the *Poscons* method (Figures 3 and 4, Supplementary File S2). Figures 3 and 4 provide the European-American and Asian DAF distributions for H and HR sites as predicted by the *Turnover* method, as well as for C sites, S sites and I sites.

Although TFBSs may be up to 25 nucleotides in length, they generally have a “core” region that is most preserved among various instances of the site in the genome and usually considered to be the most important for the physical interaction between the TF and DNA. Because mutations in the core regions are most likely to modulate TF-DNA interaction and subsequent transcription activity, we postulated that these regions may be under stronger selection than the full-length TFBSs considered above. To test this, we first determined the core region of each predicted TFBS using an information-theoretic approach (see Materials and methods). We then repeated the DAF distribution test using only the SNPs mapped to the TFBS core regions. In both European-American and Asian populations, we observed that the difference in the fraction of high frequency derived alleles between the foreground sites and the background sites was either roughly the same or larger than in the full-length TFBS analysis (Supplementary File S2). (However, this does not imply a greater statistical significance due to the substantial reduction in the number of foreground SNPs). This result provides further support for adaptive processes acting on H and HR sites.

The DAF distribution test does not take into account the genomic location of the SNPs in consideration. If the SNPs in the H and HR sites that have high derived allele frequency are largely clustered in a few genomic regions, then the larger-than-background fraction of high frequency derived alleles may be an artifact of regional (i.e. not widespread) positive selection or even selective sweep due to linkage. We compiled the chromosomal locations of all the SNPs in the H sites and observed that twenty chromosomes harbor high frequency (DAF≥90%) derived alleles. We then individually investigated each of the ten chromosomes that contribute most of the high frequency derived alleles. Overall, we observed a lack of extensive clustering of high frequency derived alleles, indicating that the results are not explained by SNP clustering (Supplementary File S3). To further ensure this, we removed the chromosome that had the most SNP clustering (chromosome 11) and repeated the DAF distribution test using sites predicted by the *Poscons* method. We found that in the Asian population, the H sites still have a significantly larger fraction of high frequency derived alleles than C sites (Fisher's exact test, P = 0.027), synonymous sites (Fisher's exact test, P = 0.019), and internal introns (Fisher's exact test, P = 0.035). The loss of significance in the European-American population is likely due to a reduction in the number of foreground SNPs.

HapMap SNPs were initially discovered based on a relatively small panel of individuals and later genotyped in larger sample populations. The probability that a SNP is ascertained is not equal for all HapMap SNPs, but rather is a function of the specific ascertainment condition that was applied. Because the HapMap project utilized a complex suite of ascertainment conditions at various phases of the SNP discovery process, some SNPs are more likely to have been identified than others[25]. This problem is partially mitigated by the Perlegen project, since the majority of SNPs, referred to as Class A SNPs, were discovered by full re-sequencing of approximately one-third of the genome in 24 individuals[26]. We repeated the above analysis using Class A Perlegen SNPs in the European-American population. We observed that HR sites have a substantially larger fraction of high frequency derived alleles than synonymous sites although not statistically significant. We hypothesized that the loss of significance may be due to differences in data size (there is a ∼75% reduction in the number of SNPs from HapMap to Perlegen). To test this, we generated random samples of the HapMap data of size equal to the Perlegen data and repeated the DAF distribution test for each sample. We found that only ∼25% of the samples displayed statistical significance (Supplementary File S2), which indicates that the lack of strong statistical significance in the Perlegen analysis could very well be explained by its smaller sample size. We repeated the sampling with increasing sample size and found a monotonic increase in the fraction of samples that show significant difference between foreground and background in the high frequency range of the corresponding DAF distributions (Supplementary File S2).

### Functional enrichment analysis of genes with high DAF regulatory SNPs

We next sought to determine if there are certain functional classes of genes for which positive selection on proximal promoter TFBSs is especially pronounced. First, we compiled the list of genes associated with each TFBS SNP from HapMap that has a high frequency derived allele. We then used the tool DAVID on the gene list (http://david.abcc.ncifcrf.gov/) to perform a functional enrichment analysis[27]. For the European-American population, the two most significantly enriched biological processes in the gene lists based on the H high frequency derived alleles (DAF≥80%) are *positive regulation of protein metabolic process* (P = 1.1 * 10^−5^) and *developmental process* (P = 2.1 * 10^−4^), although only the former is statistically significant after correction for multiple testing (FDR<0.05). For the Asian population the most enriched biological processes are *cell development* (P = 1.8 * 10−4) and *cell differentiation* (P = 1.0 * 10−3). The full enrichment lists are provided as supplementary File S4 and S5.

We next compared the functional enrichment results between the gene list based on the H high frequency derived alleles and the gene list based on the HR high frequency derived alleles. In the European population, although the overlap between the two gene lists is low (∼20%), we observed that the highest ranking Swiss-Prot term associated with both gene lists is *alternative splicing* (P = 4.5 * 10^−4^). We repeated the enrichment analysis after removing the overlap between the two gene lists and still observed the same result. This implies that regardless of the conservation category of the TFBS, advantageous mutations have predominantly risen to alter transcriptional activity of genes that already have multiple isoforms.

## Discussion

In this work we have performed the first genome-scale analysis of natural selection on putative TFBSs in human proximal promoters using the DAF distribution of SNPs from both the HapMap and Perlegen databases. A major limitation in the use of this SNP data, especially HapMap, is the presence of variable and sometimes unknown ascertainment biases[25]. To circumvent this issue, we avoided comparisons with standard neutral models and instead performed direct comparisons with approximately neutrally evolving sites, comprised of control promoter sites, synonymous sites, and internal intronic sites. We show that in the European-American and Asian populations, both H and HR TFBSs have a greater-than-background proportion of derived alleles in the high frequency range that is indicative of an accumulation of advantageous mutations undergoing positive selection. To ensure the robustness of this result, we: (1) utilized two separate TFBS prediction methods – *Poscons* and *Turnover*, (2) repeated the analysis on the core region of the TFBS in addition to the entire TFBS, (3) accounted for potential selective sweep effects, (4) examined CpG dinucleotide frequency bias, and (5) used three independent sets of approximately neutrally evolving control sites. It is important to note that we did not observe a similar result in the older Yoruban population. This may indicate that the positive selection we are detecting is more recent than the human-chimpanzee split, possibly after the migrations out of Africa. However, this is currently only a speculation. Further population genetic analyses of TFBS in the Yoruban population will be interesting.

We have used the DAF distribution as a heuristic to test for deviation from neutrality. However, it does not provide a quantitative estimate of the selection pressure. To this end, we attempted to apply the PRF model[19]. The primary advantage of the PRF method is that it considers the entire DAF distribution (as opposed to just the tails of the distribution) in order to infer the strength of selection. Due to complex and often unavailable ascertainment biases in the HapMap SNP dataset, we were only able to apply a naïve ascertainment correction scheme that assumed a constant ascertainment condition across all SNPs (Supplementary File S1). Unfortunately, due to this limitation and other assumptions of the PRF model (Supplementary File S1), the PRF estimates of selection pressure are artificially inflated and cannot be trusted in an absolute sense (Supplementary File S1). Soon-to-be-released sets of SNP data with no (or at least dramatically reduced) ascertainment bias, such as that from the Applera project[21], [28], will undoubtedly be useful for more accurate quantitative estimates of selection on human TFBSs. Finally, we also performed a functional enrichment analysis to show that genes whose proximal TFBSs may be undergoing recent adaptive evolution are enriched for functions related to protein metabolism and development.

At least two major issues must be considered when adapting standard tests of neutrality, such as the DAF distribution analysis, to *cis*-regulatory DNA. First, there is a concern regarding the specificity of current approaches for the prediction of human TFBSs. Binding motifs are not informative enough to distinguish functional sites from non-functional ones based on sequence content alone. For instance, using the set of PWM match p-values for all 235 PWMs on every position and strand of every promoter in our set, when we compute the false discovery rates or the q-values[29], our p-value threshold of 2e-05 corresponds to a false discovery rate of 39%. In an alternative analysis, at the specified match threshold, the total number of binding sites in our set of promoters, before collapsing the overlaps, is 240160, while in a set of artificially generated sequences of the same length, there were 142202 matches. This corresponds to an estimated false positive rate of 59%. However, these estimates of false positive rate may be exaggerated, because functional binding is determined by not only the cis element but several other genomic and epigenomic markers in the vicinity and the proximal promoters are enriched for these additional markers. In other words, a high scoring cis element is more likely to be functional in a ‘favorable’ chromatin region than an identical cis element in an unfavorable region. To minimize the false positives, we have utilized highly stringent thresholds in the TFBS prediction methodology, and have restricted our analysis to proximal promoters. Despite the potential for noise in the foreground data, the observed signals for positive selection are encouraging. Second, it is not clear how to best select non-coding neutrally evolving sequence. The advantage of using synonymous sites, in the case of coding region analysis, is two-fold. They are thought to be undergoing (approximately) neutral evolution and they occur in the same genomic locus as the test region (i.e. non-synonymous sites). We have attempted to select control promoter regions in order to mimic these advantages as closely as possible. Furthermore, we have also repeated the tests relative to synonymous sites and internal introns and showed similarly significant results.

To test the role of the non-coding portion of the genome in determining the phenotypic variability between individuals and among species, several recent studies have investigated natural selection in these regions. However, because the non-coding region is a heterogeneous mix of a wide variety of functional elements, a functional-class-specific investigation promises a more detailed view of natural selection. While Chen and Rajewsky[12] took the first step in this direction by investigating selection on putative miRNA target sites, here we extend this study to predicted TFBSs in proximal promoters. In contrast to the Chen and Rajewsky study, which found evidence for purifying selection acting on both conserved and non-conserved miRNA target sites in the 3′ UTR, we find evidence for recent positive selection on both human-specific as well as primate-specific TFBSs. This is perhaps best explained by binding-specificity differences between TFs and miRNAs. Because miRNA targeting has a stringent requirement for consecutive base pairing at the 5′-end of the miRNA[30], even single nucleotide interruptions to this base pairing can dramatically affect the efficacy of miRNA binding, and consequently be deleterious[31], [32]. On the contrary, TF binding motifs are substantially more degenerate and therefore likely to be more amenable to nucleotide changes that modulate TF activity to varying degrees. Finally, consistent with[12], in the more deeply conserved TFBSs (conserved among human, rhesus and mouse) we find a trend consistent with purifying selection, presumably because these sites play a role that is common to most mammals. This latter trend was, however, not statistically significant, likely due to the insufficient number of SNPs. Future studies of natural selection in regulatory elements will greatly benefit from: (i) larger sets of SNP data with minimal ascertainment biases from larger and more diverse populations, (ii) high-throughput experiments to determine regulatory elements, and (iii) novel statistical inference techniques that incorporate epistatic interaction between SNPs and relax some of the assumptions made by current models.

## Supporting Information

Supplementary File S1 Additional methods and results
(0.06 MB DOC)

Supplementary File S2 Provides the data underlying most of the results including the DAF analysis.
(0.69 MB XLS)

Supplementary File S3 Provides the chromosome clustering analysis of high DAF SNPs.
(14.09 MB XLS)

Supplementary File S4 Provides the GO enrichment results for the high DAF SNPs in European-American population.
(0.03 MB TXT)

Supplementary File S5 Provides the GO enrichment results for the high DAF SNPs in Asian population.
(0.03 MB TXT)
