# Supplementary material for: Genome-Wide Analysis of Natural Selection on Human Cis-Elements
Source: PLoS One. 2008 Sep 10;3(9):e3137. doi: 10.1371/journal.pone.0003137 (PMC2522239; doi:10.1371/journal.pone.0003137)
Supplement: Supplementary File S1 — Additional methods and results (0.06 MB DOC) [file pone.0003137.s001.doc]

**DAF distribution test of neutrality**

Errors during cellular DNA replication or repair give rise to point mutations. A mutation creates a new allele, which after achieving a population frequency of at least 5%, is referred to as the derived allele (the original non-mutated allele is known as the ancestral allele). Various evolutionary forces such as natural selection and random genetic drift can act upon a derived allele to change its frequency in a population over time. Some derived alleles do not affect the fitness (an individual’s ability to survive and productively reproduce) of the organism and the fate of these alleles in the population is determined solely by neutral evolution or random genetic drift (fluctuations due to stochastic sampling effects). However, the derived alleles that do affect fitness are subject to natural selection. Briefly, assuming little or no genetic drift, if a derived allele is deleterious to the organism, then it will be selected against and ultimately removed from the population, but if it is advantageous, then it will be selected for and eventually fixed in the population. According to Kimura’s original neutral theory of evolution [1] and Ohta’s revised nearly neutral (slightly deleterious) model [2], the latter is quite rare relative to neutral and deleterious derived alleles. For a more detailed discussion of the various evolutionary pressures on derived alleles, we refer the reader to [3].

Theoretical work in population genetics during the last several decades provides a model for the expected number of derived alleles found in a population as a function of frequency – DAF distribution [4]. Under neutral evolution, most derived alleles are expected to be at a low frequency. This is because the fixation (frequency = 1) probability of a neutral derived allele is equal to the initial frequency of the derived allele in the population (1/N, where N is the number of total gene copies in the population), which is increasingly small as N increases. Therefore, most neutral derived alleles are likely, by chance, to not move toward fixation, but rather, toward extinction (low frequency range). If some of the derived alleles are deleterious, then the proportion of derived alleles in the low frequency range will be even higher relative to neutral because negative selection will apply additional pressure to eradicate these alleles from the population. On the other hand, if some of the derived alleles are beneficial, then the proportion of derived alleles in the high frequency range will be significantly higher relative to neutral because positive selection will rapidly lead these alleles toward fixation in the population. It must be noted that factors other than selection pressures, such as sudden and dramatic change in population size or migratory patterns, can alter the DAF distribution relative to neutral expectation. However, if these factors can be assumed to be negligible, then this model provides a method of testing whether the frequency distribution of a set of derived alleles deviates from neutral expectation enough to conclude that the alleles are under selection. We will refer to this test as the DAF distribution test of neutrality.

**Poisson random field model**

Various so-called “tests of neutrality” (such as the DAF distribution test discussed above) have been developed to detect natural selection on a particular gene or genomic location [5]. However, these tests are often qualitative and only provide the directionality of selection. In the early 90s, Sawyer and Hartl provided a mathematical framework with which to determine quantitatively the intensity of selection on a particular gene, which they applied to the *Adh* locus in the *Drosophila* genome [6]. This is referred to as the Poisson Random Field (PRF) model. Owing to the recent availability of whole genome sequences and genome-wide human polymorphism data, it has become increasingly tractable to perform genome-wide scans for signatures of selection. The PRF has been applied to estimate the intensity of selection on synonymous and non-synonymous sites throughout mitochondrial and nuclear genomes of a variety of species, including human [7-15]. Very recently, Chen and Rajewsky used the PRF, among other techniques, to provide evidence for strong negative selection (even stronger than on non-synonymous coding sites) on miRNA target sites [16]. Due to the potentially wide range of applications of, and opportunities for theoretical extensions to, the PRF model, it is an increasingly important mathematical framework for quantitative geneticists. For a detailed tutorial of the PRF model, we refer the reader to [17].

The original PRF makes the following assumptions: (1) mutations arise according to a Poisson process, (2) each mutation occurs at a new site (infinite-sites, irreversible), and (3) each mutation follows an independent Wright-Fisher process with selection (no linkage).

According to the PRF framework, the number of polymorphic sites in a population with DAF between x_1_ and x_2_ is defined by a Poisson process with mean given by:

$\int_{x_{1}}^{x_{2}} g(x) dx$

where

$$\begin{matrix} g(x) =\theta\frac{1-e^{-2\gamma(1-x)}}{(1-e^{-2\gamma}) x(1-x)} \\ \end{matrix}$$

and where $\theta$ is the per-locus mutation rate (scaled by effective population size) and $\gamma$(*2Ns*) is the selection coefficient.

ß

In a sample of size *n*, the expected number of sites with *i* (which ranges from 1 to *n-1*) copies of the derived allele is then:

$$F(i) = \int_{0}^{1} g(x) P(i|x) dx = \int_{0}^{1} g(x) \left(\begin{matrix} n \\ i \end{matrix}\right) x^{i} (1-x)^{n-i} dx$$

A detailed derivation of both *g(x)* and *F(i)* are provided in [6, 18] and a recent review of these derivations is provided in[17] .

Consider sample data, such as HapMap or Perlegen data, *X = (X_1_, X_2_, X_3_, … , X_n-1_)* where *X_i_* is the observed number of sites with *i* copies of the derived allele out of *n* genotyped chromosomes. Each random variable *X_i_* is assumed to follow an independent Poisson distribution (and therefore, *X* is referred to as a Poisson Random Field) with mean equal to *F(i)*. This framework yields the probability of observing *x_i_* sites that have *i* copies of the derived allele (and *n-i* copies of the ancestral allele):

$$P(X_{i} = x_{i}|\theta, \gamma) = \frac{e^{-F(i)}F(i)^{x_{i}}}{x_{i}!}$$

where $\theta$ = *2N*$\mu$ is the mutation rate per site.

Since the *X_i_*‘s are assumed to be independent, the probability of observing *X = (X_1_, X_2_, X_3_, … , X_n-1_)* is given as:

$P(X) = L(\theta, \gamma) = \prod_{i=1}^{n-1} P(X_{i} = x_{i}|\theta, \gamma)$

This framework yields maximum likelihood estimates of selection pressure and mutation rate for the observed data.

We first fit the PRF to HapMap data in the European-American population. The maximum-likelihood estimate of the selection coefficient ($\gamma$) is +2.99 for synonymous sites, +3.54 for F_H sites, and +3.80 for F_HR sites. The probability that a SNP is ascertained is also dependent on its allele frequency in the population. Since rarer SNPs are less likely to be identified in a collection of individuals that is substantially smaller than the population size, the DAF distribution will have an artificially increased proportion of alleles in the intermediate frequency range (common SNPs). This ascertainment bias is especially pronounced in the HapMap data because the HapMap’s SNP discovery panel was often extremely small[19] . Since the PRF uses the entire DAF distribution to estimate selection pressure, we speculated that the unexpectedly high estimate of $\gamma$for synonymous sites was largely due to this ascertainment bias in the HapMap data. Expectation-Maximization algorithms have been developed to correct for ascertainment bias and determine the maximum likelihood estimate of the true DAF distribution[20] . The power of these methods is dependent upon the level of accuracy in the ascertainment conditions applied to each SNP. Unfortunately, the HapMap phase II ascertainment conditions are either too complex or unavailable. We considered the utility of a recently published subset of HapMap data for which the ascertainment conditions are known and uniform[21] . However, this, as well as the Perlegen, sample is lacking in power for our analysis because of its size. In light of this, we employed the ascertainment correction scheme in [22] with the naïve assumption of a constant ascertainment condition (single-hit in ascertainment sample of size 10) across all SNPs. We fit the PRF to the reconstituted DAF distributions and estimated $\gamma$as +1.96 for synonymous sites, +3.2 for F_H sites, and +3.8 for F_HR sites. Though the substantial difference between foreground sites and synonymous sites is reassuring, further analysis or conclusions are hindered by the still unrealistically high estimate of selection pressure on synonymous sites.

**References**

1. Kimura, M. Evolutionary rate at the molecular level. *Nature* **217**, 624-626 (1968).

2. Ohta, T. Slightly deleterious mutant substitutions in evolution. *Nature* **246**, 96-98

(1973).

1. Graur, D. and Li, W.H. Fundamentals of molecular evolution. *Sinauer Associates, Sunderland, MA.* (2004).

4. Fay, J.C. & Wu, C.I. Positive and negative selection on the human genome. *Genetics* **158**, 1227-1234 (2001).

5. Biswas, S. and Akey, J.M. Genomic insights into positive selection. *Trends Genet* **22**, 437-446 (2006).

6. Sawyer, S.A. and Hartl, D.L. Population genetics of polymorphism and divergence. *Genetics* **132**, 1161-1176 (1992).

7. Akashi, H. Inferring weak selection from patterns of polymorphism and divergence at “silent” sites in *Drosophila* dna. *Genetics* **139**, 1067-1076 (1995).

8. Nachman, M.W. Deleterious mutations in animal mitochondrial dna. *Genetica* **102/103**, 61-69 (1998).

9. Rand, D.M. and Kann, L.M. Mutation and selection at silent and replacement sites in the evolution of animal mitochondrial dna. *Genetica* **102/103**, 393-407 (1998).

10. Akashi, H. Inferring the fitness effects of dna mutations from polymorphism and divergence data: Statistical power to detect directional selection under stationarity and free recombination. *Genetics* **151**, 221-238 (1999).

11. Weinreich, D.M. and Rand, D.M. Contrasting patterns of nonneutral evolution in proteins encoded in nuclear and mitochondrial genomes. *Genetics* **156**, 385-399 (2000).

12. Bustamante, C.D., Nielsen, R., Sawyer, S., Olsen, K.M., Puruggannan, M.D. et al. The cost of inbreeding in *Arabidopsis*. *Nature* **416**, 531-534 (2002).

13. Sawyer, S.A., Kulathinal, R.J., Bustamante, C.D. and Hartl, D.L. Bayesian analysis suggests that most amino acid replacements in *Drosophila* are driven by positive selection. *Journal of Molecular Evolution* **57**, S154-S164 (2003).

14. Bartolome, C., Maside, X., Yi, S., Grant, A.L., and Charlesworth, B. Patterns of selection on synonymous and nonsynonymous variants in *Drosophila* miranda. *Genetics* **169**, 1495-1507 (2005).

15. Bustamante, C.D., Fledel-Alon, A., Williamson, S., Nielsen, R., Hubisz, M.T. et al. Natural selection on protein-coding genes in the human genome. *Nature* **437**, 1153-1157 (2005).

16. Chen, K. and Rajewsky, N. Natural selection on human microRNA binding sites inferred from SNP data. *Nat Genet* **38**, 1452-1456 (2006).

1. Sethupathy, P. & Hannenhalli, S. A Tutorial of the Poisson Random Field Model in Population Genetics. *Advances in Bioinformatics* **doi:10.1155/2008/257864** (2008).

18. Hartl, D.L., Moriyama, E.N. & Sawyer, S.A. Selection intensity for codon bias. *Genetics* **138**, 227-234 (1994).

19. Clark, A.G., Hubisz, M.J., Bustamante, C.D., Williamson, S.H. & Nielsen, R. Ascertainment bias in studies of human genome-wide polymorphism. *Genome Res* **15**, 1496-1502 (2005).

20. Nielsen, R., Hubisz, M.J. & Clark, A.G. Reconstituting the frequency spectrum of ascertained single-nucleotide polymorphism data. *Genetics* **168**, 2373-2382 (2004).

21. Keinan, A., Mullikin, J.C., Patterson, N. & Reich, D. Measurement of the human allele frequency spectrum demonstrates greater genetic drift in East Asians than in Europeans. *Nat Genet* **39**, 1251-1255 (2007).

22. Nielsen, R., Hubisz, M.J. & Clark, A.G. Reconstituting the frequency spectrum of ascertained single-nucleotide polymorphism data. *Genetics* **168**, 2373-2382 (2004).
